# Cytosolic Ku70 regulates Bax-mediated cell death

**DOI:** 10.1007/s13277-016-5202-z

**Published:** 2016-08-03

**Authors:** Manila Hada, Chitra Subramanian, Phillip C. Andrews, Roland P. S. Kwok

**Affiliations:** 1 Department of Biological Chemistry, University of Michigan Medical School, Ann Arbor, MI USA; 2 General Surgery, University of Michigan Medical School, Ann Arbor, MI USA; 3 Obstetrics and Gynecology, University of Michigan Medical School, 1150 West Medical Center Drive, Ann Arbor, MI 48109 USA

**Keywords:** apoptosis, histone deacetylase

## Abstract

The first known function of Ku70 is as a DNA repair factor in the nucleus. Using neuronal neuroblastoma cells as a model, we have established that cytosolic Ku70 binds to the pro-apoptotic protein Bax in the cytosol and blocks Bax’s cell death activity. Ku70-Bax binding is regulated by Ku70 acetylation in that when Ku70 is acetylated Bax dissociates from Ku70, triggering cell death. We propose that Ku70 may act as a survival factor in these cells such that Ku70 depletion triggers Bax-dependent cell death. Here, we addressed two fundamental questions about this model: (1) Does all Bax, which is a cytosolic protein, bind to all cytosolic Ku70? and (2) Is Ku70 a survival factor in cells types other than neuronal neuroblastoma cells? We show here that, in neuronal neuroblastoma cells, only a small fraction of Ku70 binds to a small fraction of Bax; most Bax is monomeric. Interestingly, there is no free or monomeric Ku70 in the cytosol; most cytosolic Ku70 is in complex with other factors forming several high molecular weight complexes. A fraction of cytosolic Ku70 also binds to cytosolic Ku80, Ku70’s binding partner in the nucleus. Ku70 may not be a survival factor in some cell types (Ku70-depletion less sensitive) because Ku70 depletion does not affect survival of these cells. These results indicate that, in addition to Ku70 acetylation, other factors may be involved in regulating Ku70-Bax binding in the Ku70-depletion less sensitive cells because Ku70 acetylation in these cells is not sufficient to dissociate Bax from Ku70 or to activate Bax.

## Introduction

Bax, a pro-apoptotic protein, belongs to the Bcl-2 family of proteins [1]. Activation of Bax plays an important role in both intrinsic and extrinsic apoptotic pathways. Activation of Bax can be achieved by binding to other Bcl-2 proteins, such as Bcl-2 or Bcl-Xl. Other Bcl-2 family proteins, such as that those contain only one Bcl-2 homology domain 3 (BH-3), can also activate Bax by directly binding to Bax [2]. However, Bax activity is also regulated by binding to other non-Bcl-2 proteins, such as humanin, 14-3-3, and Ku70 [3–5].

Using a neuroblastic type (N-type) of neuroblastoma (NB) cells, SH-SY5Y, we have established a model in which Bax activation is regulated by binding to the cytosolic Ku70 [6]. Ku70 was originally described as an auto-antigen [7]. However, subsequently, it was found that when dimerized with Ku80, Ku70 binds to the double strand break DNA to initiate non-homologous end-joining (NHEJ) repair [8] process. But, in a yeast two hybrid study, Ku70 was also found to be one of the factors that bind Bax [5]. The same study showed that cytosolic Ku70 binds to Bax and inhibits Bax’s pro-apoptotic activity. We and others have demonstrated that Ku70-Bax binding is regulated by acetylation of Ku70 such that when Ku70 is acetylated by the CREB-binding protein (CBP), Bax dissociates from Ku70 [9, 10]. The dissociated Bax enters mitochondria resulted in release of cytochrome C that triggers cell death. Ku70 acetylation is regulated by a class II histone deacetylase (HDAC), HDAC6. HDAC6 binds and deacetylates cytosolic Ku70 such that inhibition of HDAC6, either by using class I and II HDAC inhibitors (HDACI), such as suberoylanilide hydroxamic acid (SAHA) and trichostatin A (TSA), HDAC6-specific inhibitors, tubacin, or by depleting HDAC6 using small interfering RNA (siRNA), increases cytoplasmic Ku70 acetylation that resulted in Bax dissociation [11]. This model suggests that all cytosolic Bax binds to and is regulated by Ku70, which is inconsistent with studies demonstrating that Bax is monomeric and inactive in the cytosol [12]. This model is based on previous studies showing that in unstimulated cells, when anti-Bax antibodies (6A7) that recognizes activated Bax are used to pull down endogenous Bax, only a small amount of Bax was immunoprecipitated. Following treatment with Bax-activating compounds, like staurosporine, 6A7 was able to pull down more Bax [13], presumably activated Bax. Furthermore, gel filtration chromatography studies revealed that endogenous Bax is found in fractions at molecular weight corresponding to 29 kD proteins [14]. Thus, if Bax exists as monomers in the cytosol, how does binding of Ku70 regulate Bax’s activity? What is the stoichiometry of the binding between Ku70 and Bax? Does cytosolic Ku70 bind to all Bax in the cytosol? In this study, we have addressed two questions: first, does all Bax bind to all cytosolic Ku70? Our results show that there is only a small fraction of total cytosolic Ku70 binding to a small fraction of total Bax. The majority of Bax is monomeric. However, most cytosolic Ku70 bind to other factors forming several high molecular weight complexes. There is no free or monomeric Ku70 found in the cytosol. These results suggest that other factors may also regulate Ku70-Bax binding by restricting the availability of Ku70 that can bind to Bax.

The second question is that is Ku70 a survival factor in all cells types other than the N-type NB cells? Previously, we have shown that Ku70 plays a survival role in regulating Bax pro-apoptotic activity because depletion of Ku70 in SH-SY5Y cells results in Bax-dependent cell death [9]. However, studies have shown that, in cells, such as HeLa and HEK-293, depleting Ku70 using Ku70-specific siRNA did not induce cell death [5, 10]. These results suggest that there may be at least two cell types in terms of Ku70 regulating Bax function: one is Ku70-depletion sensitive cells, in which Ku70 acts as a survival factor (like that in SH-SY5Y cells). These cells are sensitive to Ku70 depletion, knocking down Ku70 in these cells induces cell death. The second cell type is less sensitive to Ku70 depletion (like that in HeLa cells and HEK-293 cells), in which Ku70 is not required for survival because knocking down Ku70 in these cells does not induce cell death. Here, we provided evidence that in multiple cell types (SHEP-1, ES2, A2780, and HEK-293T cells), depletion of Ku70 does not affect cell survival. Interestingly, these cells are also less sensitive to HDACI killing compared to that of N-type NB cells. Moreover, in these cells, while HDACI treatment increases cytosolic Ku70 acetylation, Bax is neither activated nor does it dissociate from Ku70. These results suggest that there may be another mechanism that regulates Ku70-Bax formation and Bax activation in Ku70-depletion less sensitive cells.

## Materials and methods

### Cell culture

HEK-293T, two ovarian cancer cell lines (ES2 and A2780) and human NB cell lines SH-SY5Y, SH-EP1, GOTO, KCN-69n, and LA1-5s were cultured in modified Eagle’s minimum essential medium (MEM) supplemented with sodium pyruvate and 10 % fetal bovine serum, and maintained at 37 °C in a humidified 5 % CO_2_ incubator.

### Cell viability assays

Cell viability was determined by either (3-(4,5-dimethylthiazol-2-yl)-2,5-diphenyltetrazolium bromide (MTT) tetrazolium reduction assay or trypan blue exclusion assay. For the MTT assay, 96-well plates were used. N-type SH-SY5Y, S-type SHEP1 human NB cell lines treated with various concentrations of SAHA, and the human ovarian cancer cell lines A2780 and ES2 were treated with varying concentrations of TSA. The viability of the cell lines was determined 24 and 48 h post-treatment by MTT assay as previously described [15]. All experiments were carried out three times with triplicates in each experiment, and the average values and standard deviations were calculated.

### Analysis of apoptosis

SH-SY5Y and HEK-293T cells grown in 60-cm plates were treated either with the solvent DMSO or with the SAHA (4 μM). Twenty-four hours after treatment, the cells were washed and suspended in annexin-binding buffer and stained with annexin V-APC and propidium iodide (PI). Induction of apoptosis was measured using a CyAn ADP Analyzer (Beckman Coulter, Inc., Indianapolis, IN) at the University of Michigan Flow Cytometry Core. The apoptotic and necrotic cells were identified based on phosphatidylserine staining with annexin on the outer leaflet of the cell membranes (apoptosis) and DNA staining by PI (necrosis and late apoptosis). Results from three independent experiments were analyzed.

### siRNA-mediated silencing and over expression of Flag-Ku70 and Flag-Ku80

For Ku70 knockdown experiments using siRNA, human NB cell lines SH-SY5Y, SH-EP1, GOTO, KCN-69n, and LA1-5s, and human ovarian cancer cell lines A2780 or ES2 cells were plated at a density of ∼2 × 10^6^ cells per 10-cm plate 24 h before transfection. The following day, the cells were transfected either with smart pool Ku70 siRNA (silencing RNA) or with the scrambled non-targeting siRNA (Dharmacon Inc.), using nucleofector kit V (Amaxa) as per the manufacturer’s instruction. Mock transfection as well as the non-targeting siRNA transfection served as controls. The level of Ku70 was determined 72 h after transfection by sodium dodecyl sulfate polyacrylamide gel electrophoresis (SDS-PAGE) using Ku70 antibodies. Either glyceraldehyde 3-phosphate dehydrogenase (GAPDH) or β-tubulin was used as a loading control. The viability of cells after knockdown was measured by counting cells using trypan blue exclusion analysis.

### Western blot analysis

The proteins were separated by SDS-PAGE, transferred to polyvinylidene difluoride (PVDF) membrane and then the blot was probed for different antibodies specific for different proteins. The following antibodies were used for western blot analyses: Ku70 (N3H10) from Santa Cruz; Ku80 (no. 2753), Bax (no. 2772S), anti-Bax antibody (6A7) (ab5714), cytochrome c oxidase subunit IV (COX IV) (no. 4844), and acetylated lysine (Ac-K-103) from Cell Signaling; and GAPDH (6C5) from Millipore and β-tubulin from Upstate. The presence of protein was visualized by using Lumigen enhanced chemiluminescence (ECL) plus PS-3.

### Immunoprecipitation

Co-immunoprecipitation of Ku70, Ku80, and Bax were performed in CHAPS buffer according to the protocol described by Sawada et al. [5] with some modifications. Cells were lysed on ice for 30 min using lysis buffer (20 mM HEPES, pH 7.5, 120 mM NaCl, and 1 % CHAPS). The extraction solution was spun twice at 3000 rpm for 10 min. The protein concentration was determined by Bio-Rad protein assay. One milligram of protein in 500 μl of lysis buffer was used for immunoprecipitation.

### Subcellular fractionation: cytosolic and mitochondrial fraction

Cells were grown in 10-cm plates to 80 % confluency. The cells were washed twice with PBS, and the cells were suspended in the mitochondrial isolation buffer (5 mM HEPES pH 7.5, 210 mM Mannitol, 70 mM Sucrose, 1 mM EDTA) in 1 million cells/100 μl of buffer. The cells were swollen on ice for 30 min and were dounced in a dounce homogenizer 50 times. The lysed cells were spun at 1500 rpm for 5 min at 4 °C. The supernatant was re-spun at 3000 rpm for 10 min. The remaining supernatant, which contained cytosolic and mitochondrial fractions, was re-spun at 10,000 g for 20 min at 4 °C. The supernatant was collected as the cytosolic fraction and the pellet was collected as mitochondrial fraction. The mitochondrial pellet was washed once with the mitochondrial isolation buffer. Equal amounts of cytosolic and mitochondrial protein were separated by SDS-PAGE. The purity of the mitochondrial and cytosolic fractions was determined by immunoblotting with COX IV and β-tubulin, respectively.

### DC4 cross-linking

DC4 cross-linker was used in our cross-linking analyses [16]. Cross-linking was carried out in a buffer containing 100 mM HEPES (pH 7.5) and 150 mM NaCl. Each cross-linking reaction contained 10–15 μg of cytosolic or nuclear proteins with 0, 0.1, 0.2, 0.4, 0.8, or 1.6 mM of DC4 per sample. The cross-linking reaction was carried out on ice for 30 min. The reaction was stopped by SDS-loading buffer. Cross-linked samples were separated by 10 % SDS-PAGE, and the blot was probed with Ku70, Ku80, or Bax antibodies.

### Gel filtration

Gel filtration chromatography was carried out using a Bio-Rad Biologic DueFlow system with a Superdex 200 HR 10/30 column at a flow rate of 0.5 ml per min. One milligram of cytosolic extracts was injected into the column. The running buffer was the same as the extraction buffer containing 20 mM HEPES (pH 7.5), 120 mM NaCl, and 1 % CHAPS. Half a milliliter fraction was collected. Twenty microliters of each fraction was separated by SDS-PAGE, and the blot was probed with Ku70, Ku80, or Bax antibodies.

## Results

### Cytosolic Ku70 forms high molecular weight complexes, while Bax is found to be mainly monomeric

**Fig. 1:**
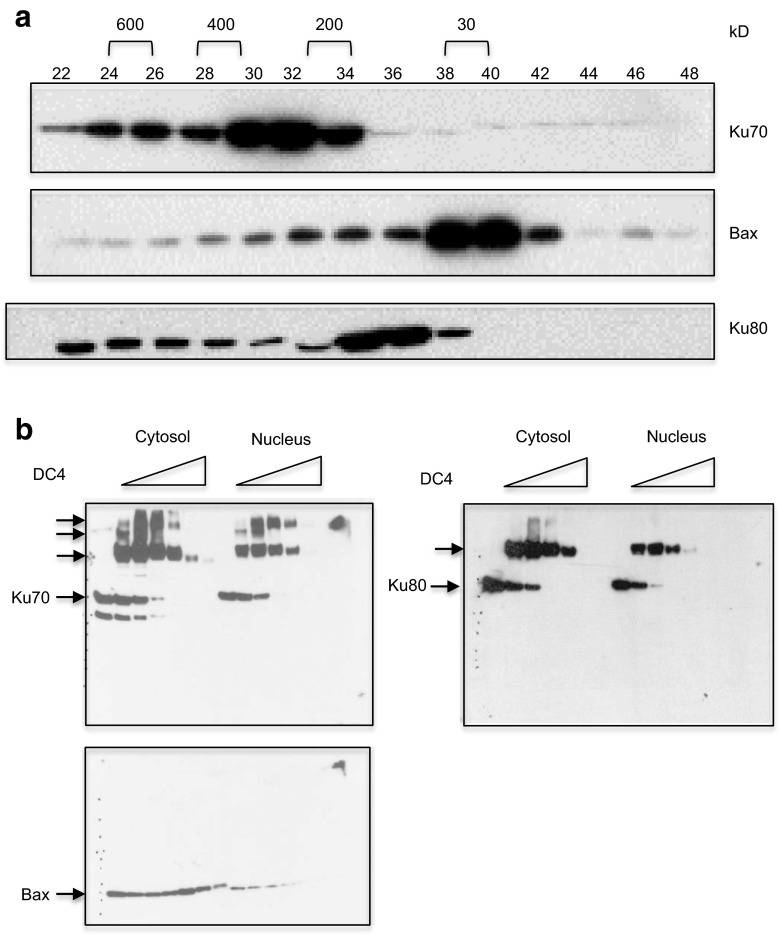
The majority of cytosolic Ku70 is found in high molecule weight complexes. **a** Cytosolic SH-SY5Y cell extracts were analyzed by Superdex 600 HR 10/30 gel filtration chromatography. Twenty microliter of each fraction (0.5 ml) was separated by sodium dodecyl sulfate polyacrylamide gel electrophoresis (SDS-PAGE), and the blot was probed with Ku70, Bax, or Ku80 antibodies. **b** Cytosolic extracts were cross-linked using DC4 (0, 0.1, 0.2, 0.4, 0.8, 1.6 mM) as shown. Cross-linked proteins were separated by 10 % SDS-PAGE, and the blot was probed with Ku70, Ku80, or Bax antibodies

Gel filtration chromatograph results show that the majority of cytosolic Ku70 in SH-SY5Y cells was found in fractions corresponding to high molecular weight complexes. In contrast, Bax was found in fractions at around 29kD (Fig. 1a), as previously described [14]. There was little overlap between Ku70 and Bax. These results indicate that not all cytosolic Ku70 interacts with all Bax in the cytosol; there is only a small number of fractions found to contain both Ku70 and Bax. Furthermore, the results indicate that the majority of Ku70 was found in fractions corresponding to large molecular complexes. Interestingly, the pattern of Ku80 in the gel filtration chromatography was different from that of Ku70. The majority of Ku80 was found in fractions corresponding to 200 kD.

To investigate whether Ku70 is found in one high molecular weight complex, as shown Fig. 1a, or in multiple complexes, we have conducted a cross-linking experiment using a cross-linker, DC4 [16]. We cross-linked cytosolic extracts and nuclear extracts of SH-SY5Y cells with various concentrations of DC4 as shown in Fig. 1b. The cross-linked complexes were separated by SDS-PAGE, and the blot was probed with Ku70, Ku80, or Bax antibodies. Cytosolic Ku70 was found in at least three high molecular weight complexes, while nuclear Ku70 was found in only two high molecular weight complexes (Fig. 1b). One of the Ku70 containing complexes (in the cytosol and in the nucleus) also contained Ku80. Most importantly, however, there was no monomeric Ku70 or Ku80 in the cytosol and in the nucleus because monomeric Ku70 and Ku80 disappeared in the SDS-PAGE immunoblots when increasing amounts of DC4 were used. The Bax cross-linking result was also consistent with the model in that only a very small fraction of Bax was complexed with Ku70; increasing cross-linker concentration did not seem to reveal a Bax-Ku70 complex due to low level of this complex.

### Ku80 mainly forms a complex with Ku70 in cells

**Fig. 2:**
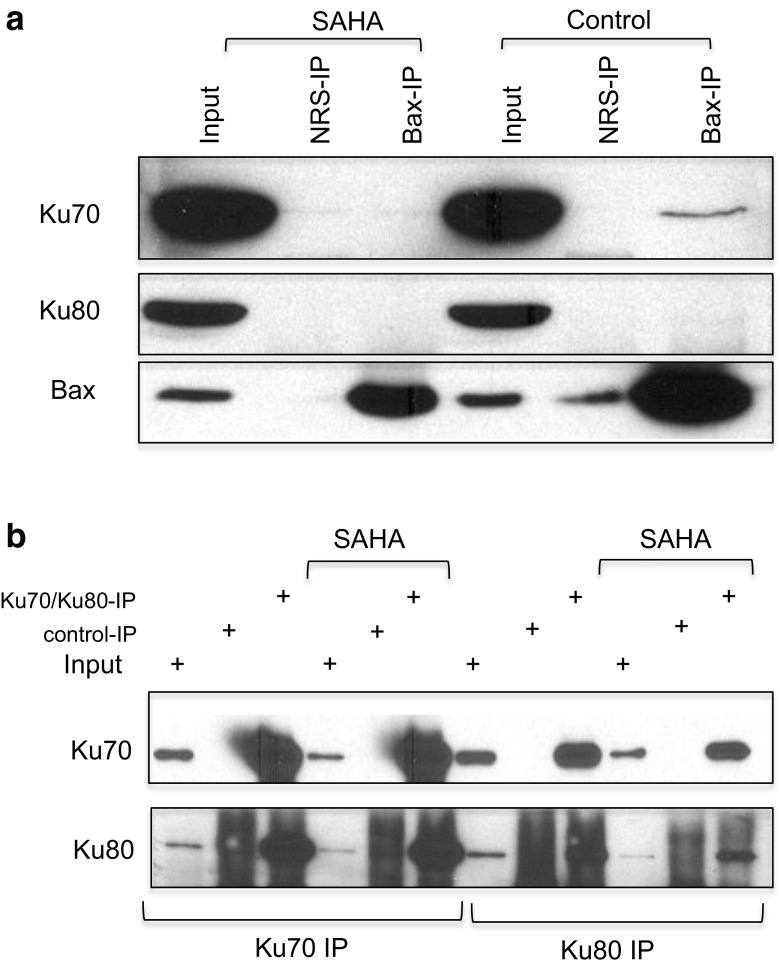
No interaction between Bax and Ku80. **a** SH-SY5Y cells were treated with suberoylanilide hydroxamic acid (SAHA) (4 μM) for 48 h. Control cells received the same volume of DMSO. Cytosolic extracts were immunoprecipitated using an anti-Bax antibody. Normal rabbit serum (NRS) was used as a negative control. The immunoprecipitated complex was separated by sodium dodecyl sulfate polyacrylamide gel electrophoresis (SDS-PAGE), and the blot was probed with Ku70, Ku80, or Bax antibodies. **b** SH-SY5Y cells were treated with SAHA (4 μM) for 48 h. Control cells received the same volume of DMSO. Cytosolic extracts were immunoprecipitated using Ku70 or Ku80 antibodies. Normal mouse serum for Ku70 immunoprecipitation or normal rabbit serum for Ku80 immunoprecipitation was used as an immunoprecipitation control, respectively. The immunoprecipitated complex was separated by SDS-PAGE, and the blot was probed with Ku70 or Ku80 antibodies

Ku80 when analyzed by gel filtration chromatography was found in fractions close to the fractions containing Bax (Fig. 1a). Ku80 only overlapped with Ku70 in a few fractions. These results suggest that Bax may interact with Ku80 in cells. However, our immunoprecipitation results shown in Fig. 2a did not support this hypothesis, at least in SH-SY5Y cells. Our results show that Ku70 was precipitated by anti-Bax antibody in cytosolic extracts of control SH-SY5Y cells but not in cytosolic extracts treated with SAHA (4 μM) for 48 h. In contrast, Ku80 was not precipitated by the anti-Bax antibody in SAHA-treated or non-treated cytosolic extracts. These results suggest that despite the gel filtration chromatography results showing that fractions containing Ku80 were closer to the fractions containing Bax, these two proteins might not interact with each other in these cells.

Gel filtration chromatography showed that the elution patterns of Ku70 and Ku80 were very different. There was no significant overlap between Ku70 and Ku80 (Fig. 1a). However, in the cross-linking study, one of the Ku70-containing complexes was Ku80 positive when probed with a Ku80 antibody. We then asked whether Ku70 and Ku80 interact with each other in the cytosol of SH-SY5Y cells. We immunoprecipitated Ku70 or Ku80 using Ku70 or Ku80 antibodies in the cytosolic extracts of SH-SY5Y cells with or without 48 h SAHA (4 μM) treatment. Normal mouse serum (NMS) or normal rabbit serum (NRS) was used as a negative control for Ku70 antibody or Ku80 antibody, respectively. Immunoprecipitates were separated by SDS-PAGE, and the blot was probed with Ku70 or Ku80 antibodies. Figure 2b shows that Ku70 or Ku80 immunoprecipitation pulled down Ku80 or Ku70, respectively, in cytosolic extracts that were either treated or untreated with SAHA. These results indicate that Ku70 interacted with Ku80 in the cytosol of SH-SY5Y cells, and this interaction was not affected by SAHA treatment.

### Ku70 depletion induces apoptosis specifically in SH-SY5Y cells but not in other cancer cell types

**Fig. 3:**
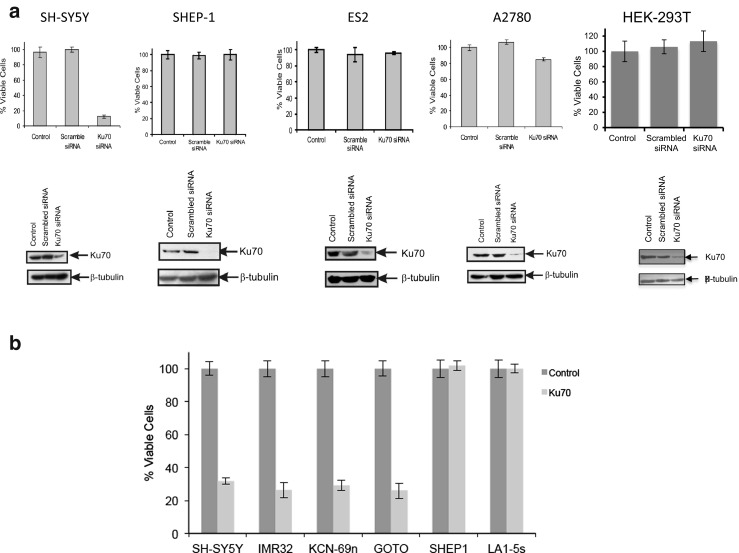
Ku70 depletion reduces cell viability in SH-SY5Y cells but not in SHEP-1, ES2, A2780, or HEK-293T cells. **a** Cells received two sequential transfections of Ku70-specific small interfering RNA (siRNA). Scrambled siRNA was used a negative control. Two days after the last transfection, cell viability was determined by trypan blue exclusion assay. The degree of Ku70 depletion was determined by SDS-PAGE using the anti-Ku70 antibody. β-Tubulin was used as a loading control. **b** Cells received two sequential transfections of Ku70-specific siRNA. Scrambled siRNA was used as a negative control. Two days after the last transfection, cell viability was determined by trypan blue exclusion assay

To address whether Ku70 depletion induces cell death in cell types other than SH-SY5Y cells, we knocked down Ku70 using Ku70 siRNA in several cancer cell lines: N-type NB cells (SH-SY5Y), stromal-type (S-type) NB cells (SHEP-1), ovarian cancer cells (A2780 and ES2), and HEK-293T. Ku70 siRNA or scrambled siRNA was transfected in various cell lines. Forty-eight hours after transfection, the cell viability was determined by trypan blue exclusion assay. The results shown in Fig. 3a indicate that, except the NB N-type SH-SY5Y cells in which Ku70 depletion reduces cell viability, depletion of Ku70 in other cell types, such as in SHEP1, ES2, A2780, and HEK-293T cells, did not affect cell viability.

### Ku70 depletion reduces cell viability in N-type NB cells

To determine whether Ku70 depletion-induced reduction of cell viability is specific to only one type of N-type NB cells, SH-SY5Y cells, we determined cell viability in three other N-type NB cells (IMR32, KCN-69n, and GOTO) after Ku70 depletion using siRNA. We used SH-SY5Y as a positive control and SHEP-1 and LA1-5s, two S-type NB cells as negative controls. Forty-eight hours after siRNA transfection, cell viability was determined by trypan blue exclusion assay. The results shown in Fig. 3b indicate that like SH-SY5Y cells, cell viability of three other N-type NB cells was reduced by Ku70 siRNA transfection while the cell viability of the two S-type NB cells was not affected. These results suggest that not only SH-SY5Y cells but also N-type NB cells require Ku70 for survival as depletion of Ku70-induced cell death.

### Ku70, Ku80, and Bax levels are similar in all cell types

**Fig. 4:**
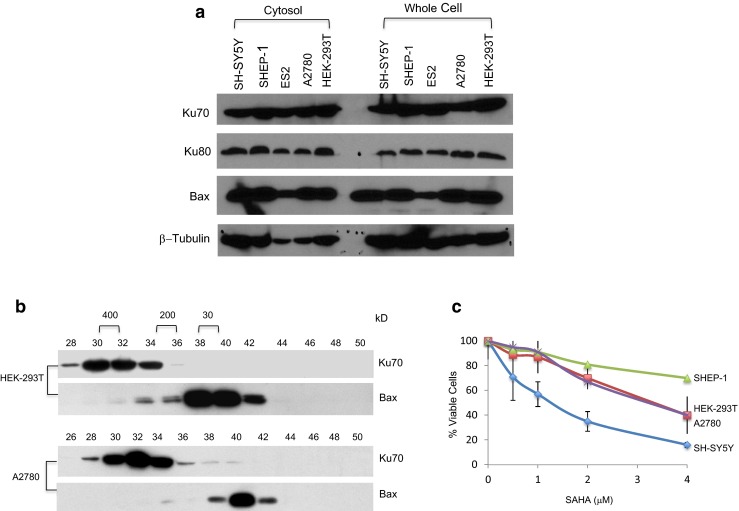
Ku70-depletion sensitive or insensitive cells have similar levels of Ku70, Ku80, or Bax, but they differ in their response to HDACI-induced cell death. **a** Cytosolic extracts or whole cell extracts of SH-SY5Y, SHEP-1, ES2, A2780, and HEK-293T cells were separated by SDA-PAGE and blotted with Ku70, Ku80, or Bax antibodies. β-Tubulin was used as a loading control. **b** Cytosolic HEK-293T or A2780 cell extracts were analyzed by Superdex 600 HR 10/30 gel filtration chromatography. Twenty microliters of each fraction (0.5 ml) were separated by SDA-PAGE, and the blot was probed with Ku70 or Bax antibodies. **c** Cells were plated into 96-well plates. One day later, they were treated with various concentrations of suberoylanilide hydroxamic acid (SAHA) as shown. There were at least three wells per concentration. Forty-eight hours following SAHA treatment, cell viability was determined by MTT assay. The results of the MTT assay were expressed as percent of DMSO-treated control (mean ± SD)

Next, we explored the differences between the Ku70-depletion sensitive cells and the Ku70-depletion insensitive cells. Here, we tested a hypothesis in which Bax requirement for cell death may be different in these Ku70-depletion insensitive cells. One possibility is that Bax may be absent in these cells so that Bax is no longer a cell death factor. Thus, depletion of Ku70 will not have any effect on cell survival. We determined the protein expression of Ku70, Ku80, and Bax in the cytosolic extracts and in the whole cell extracts in SY-SH5Y, SHEP-1, ES2, A2780, and HEK-293T cells. We separated these factors by SDS-PAGE and probed the blot with Ku70, Ku80, or Bax antibodies. β-Tubulin was used as a loading control. Figure 4a shows that, in whole cell extracts, the level of Ku70, Ku80, or Bax in all cell lines was similar, except in ES2 that had low levels of Bax. In the cytosolic extracts, the β-tubulin loading control was uneven; it was higher in SH-SY5Y and SHEP-1 cells, and to some extent also in HEK-293T cells, compared to that of ES2 and A2780 cells. However, the Bax level followed the same pattern as that of the β-tubulin loading control, being higher in SH-SY5Y, SHEP-1, and HEK-293T cells but low in other cell types. The level of cytosolic Ku70 and Ku80 was similar despite the variations in β-tubulin loading control. While we did not conduct a densitometry analysis of these bands, we felt that we can conclude from these results that there were no big variations in the level of Bax and Ku70 in the cytosolic extracts of SH-SY5Y, SHEP-1, and HEK-293T cells. These results suggest that differences in the sensitivity to Ku70 depletion were not due to the differences in the Bax or Ku70 levels.

### Ku70-depletion sensitive and insensitive cells have similar cytosolic Ku70 complex pattern

Using gel filtration chromatography and cross-linking studies, we have shown that in Ku70-depletion sensitive SH-SY5Y cells, Ku70 forms several high molecular weight complexes and that only a small fraction of Ku70 and Bax binds to each other (Fig. 1a). Here, we show that, like that of SH-SY5Y cells, Ku70-depletion insensitive cells (HEK-293T and A2780 cells) had similar patterns in gel filtration chromatography (Fig. 4b). These results suggest that there were no gross differences in terms of Ku70 or Bax distribution in the Ku70-depletion less sensitive cells compared to that of the Ku70-depletion sensitive cells.

### Different responses to SAHA treatment in different cell types

We have shown previously that SH-SY5Y cells are sensitive to HDACI treatments. Using SAHA, TSA (both class I and II HDAC inhibitors), or tubacin (an HDAC6-specific inhibitor), we have shown that HDACIs induce Ku70 acetylation, which resulted in Bax dissociation and apoptotic cell death [11, 17, 18]. Here, we explored the differences between Ku70-depletion sensitive and Ku70-depletion insensitive cells in terms of their response to HDAC inhibitor treatment. Cells were treated by 48 h with SAHA (0, 0.5, 1, 2, and 4 μM). Cell viability was determined by MTT assay. The results in Fig. 4c show that SH-SY5Y cell viability was reduced to 20 %, while the cell viability of other Ku70-depletion less sensitive cells were only reduced to 50 % (HEK-293T and A278) and to 80 % (20 % reduction for SHEP-1 cells). There were clear differences in terms of the cell viability response to HDACI treatment between Ku70-depletion sensitive and Ku70-depletion less sensitive cell types.

### HDACI induces cytosolic Ku70 acetylation but not Ku70-Bax dissociation in Ku70-depletion insensitive cell lines

**Fig. 5:**
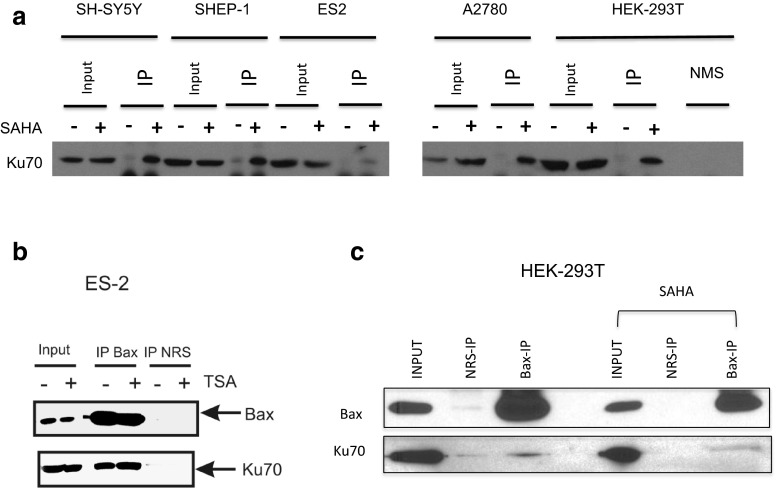
Cytosolic Ku70 is acetylated by histone deacetylase inhibitors (HDACI) in cells, and Bax did not dissociate from Ku70 in ES2 and in HEK-293T cells following HDACI treatment. **a** Cells were treated with suberoylanilide hydroxamic acid (SAHA) (4 μM) for 48 h. Control cells received the same volume of DMSO. Cytosolic extracts were immunoprecipitated using an anti-acetyl-lysine antibody. (K-103). Normal mouse serum (NMS) using HEK-293T cytosolic extracts was used as a negative control. The immunoprecipitated complex was separated by sodium dodecyl sulfate polyacrylamide gel electrophoresis (SDS-PAGE), and the blot was probed with an anti-Ku70 antibody. **b**, **c** ES2 cells were treated with TSA (10 μM) for 48 h. HEK-293T cells were treated with SAHA (4 μM) for 48 h. Cytosolic extracts were immunoprecipitated using anti-Bax antibodies. Normal rabbit serum (NRS) was used as a control. The immunocomplexes were separated by SDS-PAGE, and the blot was probed with Bax or Ku70 antibodies

We have previously shown that in Ku70-depletion sensitive cells (such as SH-SY5Y cells), HDACI treatment induces Ku70 acetylation, Bax dissociation from Ku70, and apoptotic cell death. In Fig. 4c, we show that the Ku70-depletion insensitive cells only had a partial response to HDACI treatment. Thus, we next explored whether the Ku70-Bax complex in the Ku70-depletion insensitive cells have similar response to HDACI. We first tested whether Ku70 is acetylated in Ku70-depletion less sensitive cells in response to HDACI. We treated the cells with SAHA (4 μM) for 48 h. Cytosolic extracts were immunoprecipitated using an anti-acetyl-lysine antibody (Ac-K-103). The immunoprecipitates were separated by SDS-PAGE and probed with an anti-Ku70 antibody. The results shown in Fig. 5a indicate that in both cell types, Ku70-depletion sensitive (SH-SY5Y) and Ku70-depletion less sensitive cells (SHEP-1, ES2, A2780, and HEK-293T), cytosolic Ku70 was acetylated following HDACI treatment.

We have shown previously that in SH-SY5Y cells, Ku70 acetylation, either by inhibiting HDAC6 or by depleting HDAC6, results in Bax dissociation causing apoptotic cell death [11]. Here, we asked whether Bax dissociates from Ku70 following HDACI treatment in Ku70-depletion less sensitive cells. In ES2 and HEK-293T cells, 48 h following TSA (10 μM) or SAHA (4 μM) treatment, respectively, we immunoprecipitated Bax using a Bax-specific antibody. Normal rabbit serum (NRS) was used as a negative control. The immunoprecipitates were separated by SDS-PAGE, and the blot was probed with anti-Bax antibodies or anti-Ku70 antibodies. The results in Fig. 5b, c show that the Bax antibody immunoprecipitated Ku70 in the cytosolic extracts of both cell types that were treated with or without HDACI. These results suggest that, unlike those in SH-SY5Y cells, Bax did not dissociate from Ku70 even though Ku70 was acetylated following HDACI treatment.

### Bax is not activated following HDACI treatment in Ku70-depletion insensitive cell

While the results shown in Fig. 5a demonstrate that Ku70 was acetylated following HDACI treatment, and the results in Fig. 5b, c show that Bax did not dissociate from Ku70 in the Ku70-depletion less sensitive cells, it is not clear whether Bax was activated and induced apoptosis even it still bounds to Ku70. To address these questions, we have examined several key features of cells undergoing apoptosis.

**Fig. 6:**
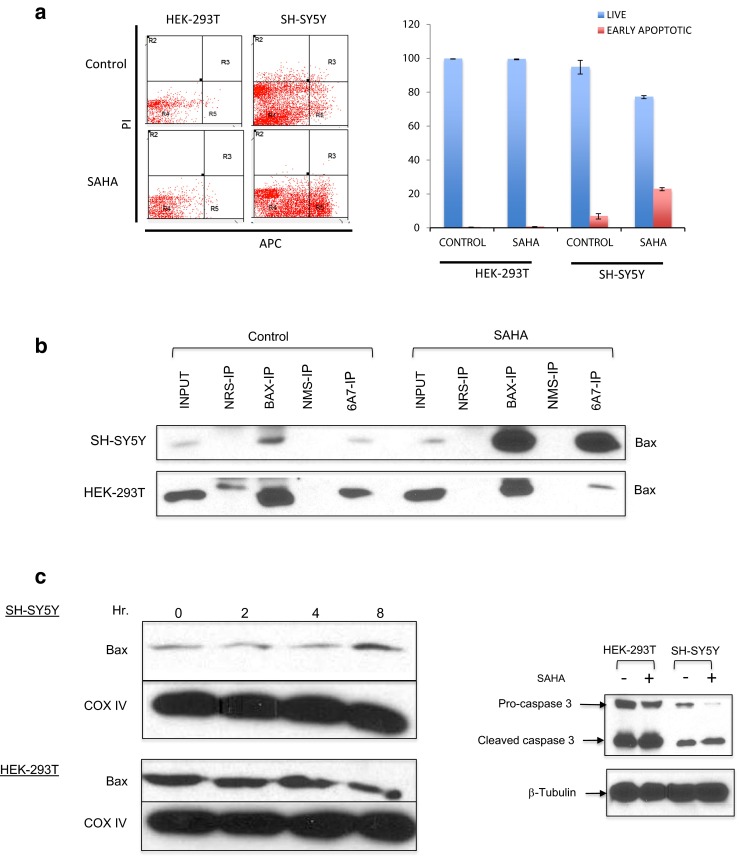
Bax is activated in SH-SY5Y cells but not in HEK-293T cells following HDACI treatment. **a**, **b**, **d** SH-SY5Y or HEK-293T cells were treated with suberoylanilide hydroxamic acid (SAHA) (4 μM) for 24 h. Control cells received only DMSO. **a** The cells were washed, suspended in annexin-binding buffer, and stained with annexin V-APC and PI. Induction of apoptosis was measured using a CyAn ADP Analyzer (Beckman Coulter, Inc., Indianapolis, IN) at the University of Michigan flow cytometry core. **b** Cytosolic extracts were immunoprecipitated using an anti-Bax antibody or an anti-activated Bax antibody (6A7). Normal rabbit serum (NRS) or normal mouse serum (NMS) was used as a control. Immunocomplexes were separated by sodium dodecyl sulfate polyacrylamide gel electrophoresis (SDS-PAGE), and the blot was probed with anti-Bax antibodies. **c** SH-SY5Y or HEK-293T cells were treated with SAHA (4 μM) for 0, 2, 4, or 8 h as shown. The mitochondrial extracts were analyzed by SDS-PAGE, and the blot was probed with anti-Bax or anti-COX IV antibodies. **d** Cytosolic extracts were analyzed by SDS-PAGE, and the blot was probed with anti-caspase 3 antibodies. β-Tubulin was used as a loading control

We first treated HEK-293T and SH-SY5Y cells with SAHA (4 μM). Twenty-four hours later, we determined the viability and apoptosis of treated cells using annexin V-APC/PI dual staining by flow cytometry. The results shown in the left panel of Fig. 6a indicate that treatment of HEK-293T cells with SAHA did not change viability, as greater than 99 % of cells were not stained by either PI or annexin V. But in SH-SY5Y cells, SAHA treatment decreased the viability of cells from 94.7 to 77.1 % (top and bottom right panels of Fig. 6a). Similarly, in HEK-293T cells, SAHA treatment did not induce any increase in annexin V positive cells. But in SH-SY5Y cells, SAHA treatment increased annexin V positive cells from 5.1 % (control) to 22.8 % (SAHA treated) with a *P* value equal to 0.014 (two-tailed *t* test, *N* = 3).

Next, we directly asked whether Bax was activated following HDACI treatment in Ku70-depletion sensitive cells (SH-SY5Y) and Ku70-depletion less sensitive cells (HEK-293T). We used an anti-Bax antibody (6A7) in an immunoprecipitation experiment. This antibody binds to the N-terminal of Bax when Bax is activated [19]. Using this method, we demonstrated that in control cells, Bax activation was very low in both SH-SY5Y cells and in HEK-293T cells (Fig. 6b). However, 24 h following SAHA (4 μM) treatment, there was a significant increase in Bax activation in SH-SY5Y cells (increase in 6A7 antibody pull down). In contrast, there was no increase in 6A7 antibody pull down in HEK-293T cells. These results suggest that, in Ku70-depletion insensitive cells, HDACI treatment did not induce Bax activation.

Another approach was to test whether Bax translocated to the mitochondria following HDACI treatment. The results in Fig. 6c show that the level of Bax in the mitochondria in SH-SY5Y cells was increased 8 h following SAHA (4 μM) treatment. In contrast, in the HEK-293T cells, SAHA treatment did not alter the level of Bax in the mitochondria. These results are consistent with the results shown in Fig. 6a, b following HDACI treatment in Ku70-depletion sensitive cells (SH-SY5Y); Bax was activated and translocated into the mitochondria. But in Ku70-depletion less sensitive cells (HEK-293T), Bax was not activated and, therefore, there was no change in Bax level.

In the last test, we studied the cleavage of pro-caspase 3, a downstream target of Bax activation, following HDACI treatment. We used an anti-caspase 3 antibody that recognizes both pro-caspase 3 and cleaved caspase 3. Both SH-SY5Y cells and HEK-293T were treated with SAHA (4 μM) for 24 h, equal amounts of cytosolic extracts from treated and untreated cells were separated by SDS-PAGE, and the blot was probed with the anti-caspase 3 antibody. β-Tubulin was used as a loading control. The results in Fig. 6d demonstrated that there was a basal cleavage of pro-caspase 3 in both cell types. However, in SAHA-treated HEK-293T cells, there was no difference in caspase 3 cleavage compared to the untreated cells. In contrast, SAHA-treated SH-SY5Y cells had markedly reduced pro-caspase 3 level and increased cleaved caspase 3. These results suggest that, as predicted, HDACI treatment of SH-SY5Y cells activated Bax, resulting in Bax translocation to the mitochondria, leading to activation of caspase 3 (cleavage of pro-caspase 3). In HEK-293T cells, HDACI treatment did not activate Bax; Bax did not translocate into mitochondria and did not cleave pro-caspase 3.

## Discussion

One of the focuses of this study was to answer a fundamental question: how much cytosolic Ku70 and Bax bind to each other in cells. A study by Sawada et al. stated that “a large proportion of the Bax population is associated with Ku70 in normal cells” [5]. However, this statement is not consistent with the published results showing that Bax is found to be inactive and monomeric in the cytosol [14]. In contrast, our results are consistent with this monomeric Bax model. We have shown that, in SH-SY5Y cells, which are Ku70-depletion sensitive, only a small fraction of cytosolic Ku70 binds to a small fraction of Bax. The majority of Bax is monomeric, and the majority of Ku70 is in complex with other factors, including Ku80, forming several high molecular weight complexes. Interestingly, there is no free Ku70 or monomeric Ku70 found in the cytosol.

We have proposed that Ku70 acts as a survival factor to protect the cells from dying of Bax-dependent apoptosis. How can a small amount of cytosolic Ku70 that binds to only a small amount of Bax protect the cell from dying? We believe that cells throughout life constantly receive stimuli, including death signals that affect cell viability. Some of the death stimuli may lead to apoptosis, while some of these small stimuli may only activate a few Bax molecules. As a survival mechanism, cells do not want to commit to die after receiving weak signals that activate only small amount of Bax. To survive these aberrant Bax activation signals, cells design a mechanism to block these signals. We believe that Ku70 may act as a survival factor, blocking the small amount of Bax that is being activated by weak cell death signals.

This model suggests that because there is only a small amount of Ku70 that binds to a small amount of Bax, additional Ku70 may be needed when more Bax is activated. However, where does the additional Ku70 come from if there is no free Ku70, and all remaining Ku70 that does not bind Bax is in complex with other factors? This model suggests another level of regulation of Ku70-Bax binding, in which Ku70 has to be released from other complexes to be available to bind to activated Bax. Studies have shown that Ku70 binds to several factors in the cytosol [20–22]. For example, the FADD-like interleukin-1β-converting enzyme (FLICE)-inhibitory protein (FLIP) is an anti-apoptotic protein blocking caspase 8 activation by death receptors [23]. However, FLIP, like Bax, also binds to Ku70 in an acetylation-dependent manner. FLIP binds to the Ku80-binding domain of Ku70. When Ku70 is acetylated at the same two lysines, K539 and K542, that regulate Bax binding, this will also dissociate FLIP, triggering FLIP polyubiquitination and degradation. This model suggests that Ku70 regulates apoptosis via the intrinsic pathway through Bax and the extrinsic pathway through caspase 8 [23]. Whether FLIP and Bax bind to Ku70 simultaneously and dissociate from Ku70 when Ku70 is acetylated at K539 and K542 is not clear.

Ku80 is a DNA-binding partner of Ku70 in the nucleus. Our results also show that Ku80 binds to Ku70 in the cytosol forming a higher molecular weight complex (Fig. 1b). This complex formation is not affected by HDACI treatment (Fig. 2a). However, unlike cytosolic Ku70, Ku80 does not bind Bax (Fig. 2a). These results are also consistent with the report by Sawada et al. showing that Ku80 does not bind Bax and that Ku70 does not bind Bax and Ku80, simultaneously [5]. Whether the Ku70-Ku80 complex can regulate Ku70-Bax binding remains to be determined.

Another issue we addressed in this study is whether Ku70 that acts as a survival factor, which we established using SH-SY5Y cells, is also applicable to other cell types. Our results show that we can clearly distinguish between two cell types based on their response to Ku70 depletion: Ku70-depletion sensitive cells, in which Ku70 depletion kills cells (such as in N-type NB cells) (Fig. 3b), and Ku70-depletion less sensitive cells (such as SHEP-1, ES2, A2780, and HEK-293T cells) (Fig. 3a), in which Ku70-depletion does not kill cells.

To investigate the mechanism by which these two cell types differ in their regulation of cell death by Ku70, we treated these two cell types with HDACI. We have previously shown that, in SH-SY5Y cells, HDACI treatment induced cytosolic Ku70 acetylation and resulted in Bax release from Ku70. The released Bax translocates into mitochondria, causing cytochrome c release, triggering cell death [17, 18]. In this study, our results show that Ku70-depletion less sensitive cells are less sensitive to HDACI-induced killing (Fig. 4c). Most importantly, cytosolic Ku70 is acetylated in these cells following HDACI treatment (Fig. 5a), but Bax is not released from Ku70. These results suggest that Ku70 acetylation regulation of Bax release in Ku70-depletion sensitive cells must be different in that in the Ku70-depletion less sensitive cells. To confirm whether Bax is activated in Ku70-depletion less sensitive cells even after HDACI-induced Ku70 acetylation, we immunoprecipitated Bax using a Bax antibody (6A7) that specifically recognizes the N-terminal of Bax when it is exposed when activated [19]. Our results show that apoptosis and Bax activation did not occur following HDACI treatment in the Ku70-depletion less sensitive cells, while apoptotic initiation (increased in phosphatidylserine) and Bax activation were found in Ku70-depletion sensitive cells following HDACI treatment (Fig. 6a, b). Furthermore, HDACI treatment induced Bax translocation to the mitochondria in Ku70-depletion sensitive cells but not in Ku70-depletion insensitive cells (Fig. 6c). Also, cleavage of pro-caspase 3, a downstream target of Bax activation, is increased in Ku70-depletion sensitive cells following HDACI treatment but not in Ku70-depletion less sensitive cells (Fig. 6d). These results suggest that the Ku70 regulation of Bax in the Ku70-depletion less sensitive cells must be different from the conventional model, in which Bax binding to the Bax-binding domain of Ku70 is regulated by the acetylation at K539 and K542 [10]. The two lysines are localized at the linker region of Ku70, not within the Bax-binding domain localized at the carboxyl terminal of Ku70. Acetylation of these two lysines induces a conformational change of the Bax-binding domain of Ku70, which resulted in Bax dissociation. Our model suggests that some other factors must be affecting the conformational change of Ku70 upon acetylation of these two lysines. These factors must be small because in gel filtration analyses, the pattern of Ku70 and Bax chromatograph is similar in HEK-293T cells compared to that of the SH-SY5Y cells (Figs. 1a and 4b). Another possibility is that the Ku70-depletion less sensitive cells have higher level of anti-apoptotic Bcl-2 family of proteins suppressing the activation of Bax and its association from Ku70 when Ku70 is acetylated.
